# Supplementary material for: Increased Rice Susceptibility to Rice Blast Is Related to Post-Flowering Nitrogen Assimilation Efficiency
Source: J Fungi (Basel). 2022 Nov 17;8(11):1217. doi: 10.3390/jof8111217 (PMC9694259; doi:10.3390/jof8111217)
Supplement: Supplementary file 1 [file jof-08-01217-s001.zip › jof-1906738-Supplemental.pdf]

ID	VARIETY NAME	ORIGIN
**SEPYA_005**	CIGALON	France
**SEPYA_007**	GINES	France
**SEPYA_009**	MANOBI	France
**SEPYA_010**	PACO	France
**SEPYA_011**	PATY	France
**SEPYA_017**	BRIO	Italy
**SEPYA_029**	Not commercial	CFR France
**SEPYA_077**	Not commercial	CFR France
**SEPYA_079**	Not commercial	CFR France
**SEPYA_096**	GLEVA	France

Table S1: List of the ten varieties used for the experiment.

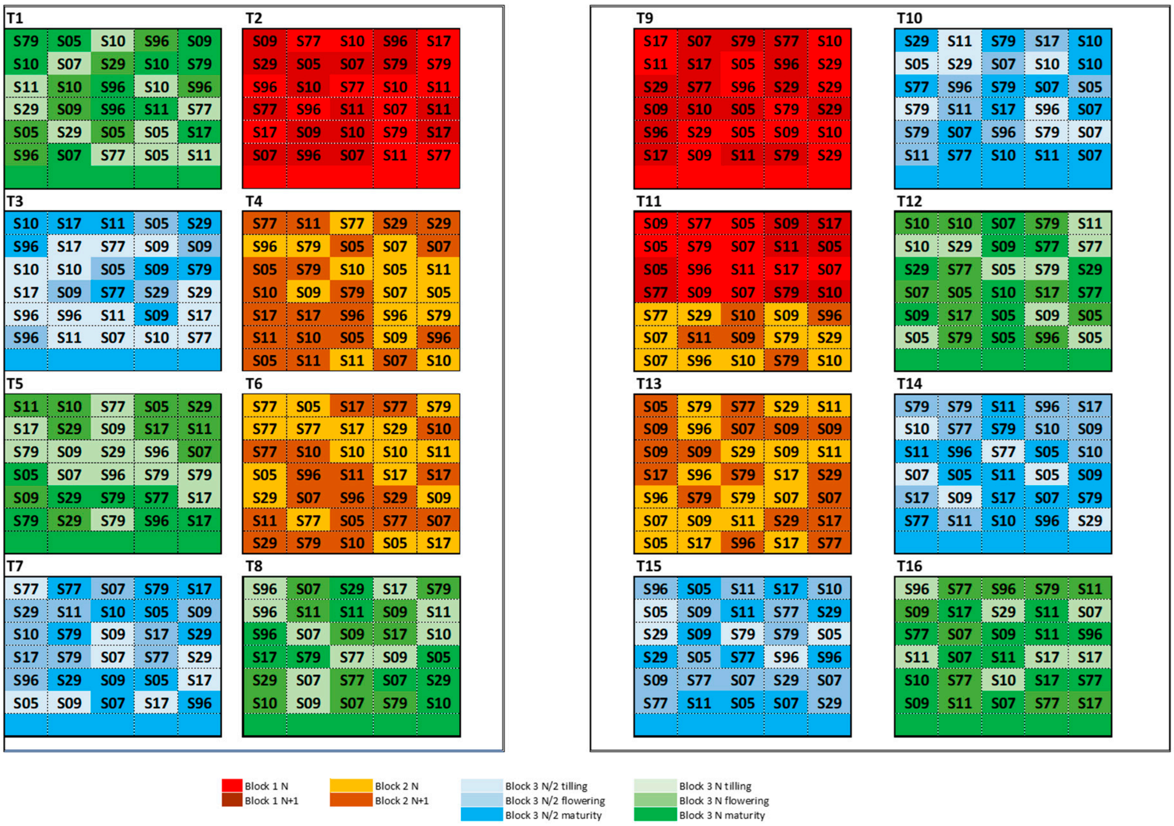

Figure S1: Greenhouse experimental design. Each box represents a pot placed on a table containing 30 pots with two plants per pot. Each large rectangle represents a greenhouse chapel. Each color represents an experimental block red for block 1, orange for block 2 and blue (N/2) and green (N) for block 3. The N and N/2 modalities are physically separated to avoid “leakage” and mixing of ni-trogen through leaching

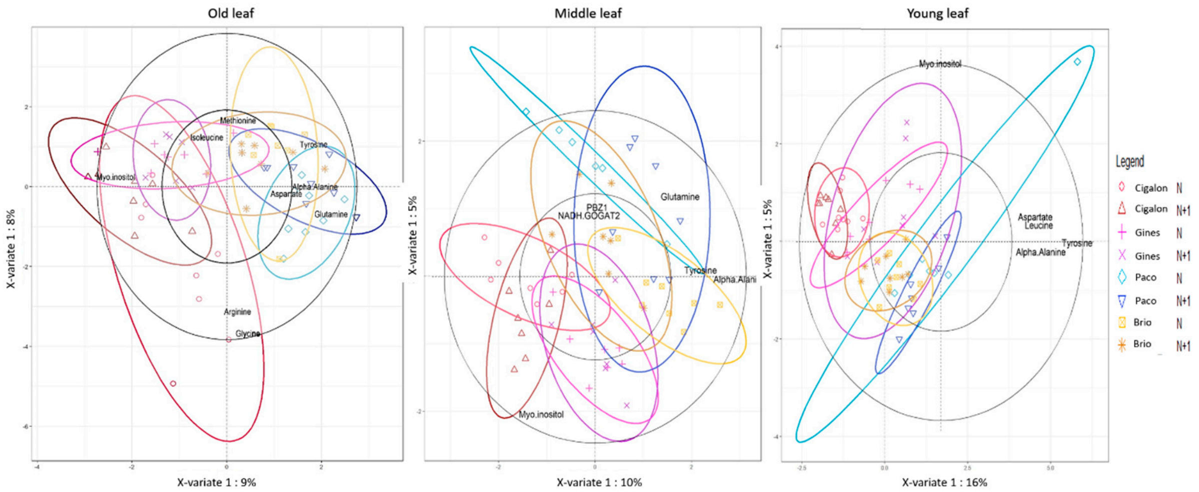

Figure S2: Combined projection of individuals and PLSDA variables calculated for each leaf level. Each PLSDA has been optimized to keep only the most discriminating variables. The best-represented variables are those that are close to the outer black circle. Projections were calcu-lated by reporting the genotype/treatment association as the discriminating group. N: no nitrogen shoot, N+1: nitrogen shoot.
